# Satisfaction with Self and External Regulation of Learning in Higher Education Students in Brazil

**DOI:** 10.3390/ijerph18115914

**Published:** 2021-05-31

**Authors:** Lucía Herrera Torres, Mara Rachel Souza-Soares de Quadros, Laura C. Sánchez-Sánchez, Tamara Ramiro-Sánchez

**Affiliations:** 1 Department of Developmental and Educational Psychology, Faculty of Science Education and Sport, University of Granada, Calle Santander, N° 1, 52071 Melilla, Spain; luciaht@ugr.es (L.H.T.); tramiro@ugr.es (T.R.-S.); 2 Centro de Convergência, Campus Morro do Cruzeiro, Universidade Federal de Ouro Preto, Ouro Preto 35400-000, Brazil; mara.rachel@ufop.edu.br

**Keywords:** self-regulation, external regulation, study techniques, teaching–learning processes, educational infrastructure, type of institution, academic year

## Abstract

The satisfaction of university students with the variables that regulate their learning provides very valuable information to improve the quality of teaching processes. The main objective of this study was to evaluate the learning of Brazilian university students, exploring both self-regulation variables, such as study techniques; and more external regulation variables, namely, satisfaction with the teaching–learning process and with the infrastructure, based on three variables: gender, the institution of higher education and the academic year of the students. To achieve this, 560 students of the Pedagogy degree were evaluated with two questionnaires: a questionnaire of satisfaction with the educational infrastructure and the teaching–learning process and a questionnaire on study techniques. Statistically significant differences were obtained, especially depending on the type of institution and the academic year. The students of private schools and earlier academic years were the ones who obtained the most satisfaction with the study techniques and with the infrastructure. Those from private centers also expressed more satisfaction with the teaching–learning processes. These results provide greater knowledge about the processes of self-regulation and external regulation of university learning and of their satisfaction with them, which can contribute to improving educational policies in Brazil.

## 1. Introduction

University learning and the many issues that it entails has become a highly relevant topic in political and academic debates [1,2]. In this framework, there have been multiple research focuses used to evaluate university learning, from the way in which students regulate their learning and how teachers perform, to the contextual and organizational factors of the institutions [3,4,5]. In recent decades, higher education institutions, with the aim of offering a favorable learning environment, have highlighted the importance of considering in-learning assessment: student satisfaction with the teaching–learning process through the teaching performance (i.e., the level of experience; teacher–student relationship; mastery of the content; pedagogical resources used, such as audiovisual, multimedia or laboratory and others) and with the educational infrastructure [6,7,8,9,10,11].

The way in which students learn is a crucial issue in the context of higher education due to its implication in aspects such as university dropouts, academic performance, difficulties in accessing the labor market or student satisfaction [12,13,14,15,16]. So much so that, as argued by Hagström and Lindberg [17], there are many investigations that have displaced the object of attention from the teaching action towards the student’s learning.

In this sense, university learning is a complex process, which is made up of a range of constructs such as learning approaches, learning styles, learning strategies, study techniques, self-regulated learning or study habits, among others [18,19,20,21,22]. Regarding this topic, the idea that students learn in different ways is defended, since they use a series of activities and strategies to apprehend the contents [23,24]. In other words, learning takes place through cognitive processing activities, affective/motivational activities, and self-regulation activities [25,26,27]. In relation to this, scientific literature has shown that the learning process is influenced by the interaction of personal and contextual factors, which condition the learning outcome [28,29]. Among personal aspects, age, personality, motivation and learning styles have been highlighted; while in contextual aspects, teaching methods, types of evaluation and academic interactions have been identified [30,31,32,33,34].

In this scenario, the concept of self-regulated learning deserves a special mention by emphasizing the way in which students see themselves as learners [35], in the sense of developing in themselves certain academic skills, such as setting objectives, selecting strategies and self-assessing their learning, that is, as active and constructive individuals [36]. This proactive stance is based on feelings and motivational beliefs and the use of metacognitive strategies [37,38]. As Pintrich and García [39] state, self-regulated learning is governed by metacognitive control (planning, monitoring and regulation of the activities carried out during learning) and by resource management strategies with respect to time and study space. In this framework, some research has shown that university students who use self-regulation strategies to learn, obtain higher academic performance as well as higher academic satisfaction [7,31]. Consequently, different studies highlight the importance of educators informing and training their students in the development of self-regulatory capacities [7,38,40,41].

In the current context, one of the most significant concerns for Educational Psychology is student behavior towards their own learning [31]. This is due to the role that the student assumes in their “learning to learn” process, which justifies the need to know, on the one hand, how students deal with the learning process [32] and, on the other hand, the teaching methodologies and teaching strategies adopted, with a view to improving learning outcomes, particularly after the Bologna Plan [40,42,43].

From this perspective, the way the teacher educates and the strategies they use influence the way the student chooses the techniques to be employed and copes with studying [23]. However, it must be emphasized that lectures still predominate in higher education, with an emphasis on content and not on competencies, centered on the figure of the teacher, which results in less reflective students [44,45,46], who use processes of superficial learning aimed at reproducing “models”, and for this reason, make use of less elaborate learning strategies, for example, the taking of nongenerative notes, without a conscious or intentional use [3,47,48]. In contrast, there are university students who use deep learning processes, which imply intrinsic motivation to learn and use more elaborate learning strategies, together with self-regulation activities [24,49], which are favored when teachers use a regulatory teaching method, that is, one which focuses especially on helping students to self-regulate their learning process [7,41].

Linked to the above, gender and academic year are some of the variables that have been frequently related to the use of learning strategies by university students [50,51,52,53,54,55]. Some research shows that women use self-regulation strategies more frequently than men [50,53,55], as well as obtaining higher scores than men in strategies of testing, elaboration, organization and metacognitive processing [52]. However, other investigations have not found differences based on gender [56,57,58,59] or find them mediated by the type of degree being studied [60] or by age [61]. With regard to the academic year, some research reports that it is students in higher academic years that make greater use of learning strategies and are more effective than those used by students in lower academic years [51,54]. There is, however, research such as that of Lynch [52], which points to a decrease in the maintenance of effort in higher academic years compared to lower ones. In the same vein, in a systematic review on the evolution of the development of learning approaches in university students, the authors concluded that the reviewed studies do not provide clear empirical evidence for the assumption that students of higher academic years or levels develop deeper or more elaborate learning strategies and approaches [62].

On the other hand, in this student-centered context, student satisfaction emerges as an extremely relevant aspect as a tool for evaluating learning in institutions of higher education (IHEs) [63,64], considered a pedagogical device [65] which is influenced by student life at the university [66]. Through student satisfaction, the motivation and permanence of the students can be achieved [67], guarantee better learning results [68], contribute to greater competitiveness [69], favor enrollment in undergraduate and postgraduate studies [70], as well as promote greater loyalty and trust [71,72], among other aspects.

Scientific literature lists a variety of factors that predict student satisfaction, including the quality of the teaching–learning process [6,73,74,75], as well as aspects related to the educational infrastructure, such as administrative staff [73,76,77,78], university facilities [65,79,80,81], location [65,78] and technological resources [66]. In this sense, educational technology should be highlighted as an important factor that influences student satisfaction, both with the educational infrastructure through the availability of technological resources and the support of information technologies [66], and with the teaching–learning process, by means of the use of technological resources by teachers [74].

Regarding satisfaction with the teaching–learning process through the teaching performance, this appears in a wide variety of studies as an important explanatory factor of overall satisfaction of university students [74,77,82,83]. Furthermore, satisfaction with the teaching–learning process has been related to the use of deeper or self-regulated learning strategies [61,84,85], as well as higher academic performance in university students [7]. Similarly, some studies highlight the influence of gender on satisfaction with the teaching–learning process, although with contradictory results [74,86]. In this way, while Poon’s [74] research carried out on university students in the United Kingdom, revealed that women showed a lower level of satisfaction with the teaching–learning process than men, in O’Driscoll’s [86] research with Irish university students, they did not find statistically significant differences based on gender. Even other research has shown that these gender differences could be mediated by the use of a deeper learning approach and the age of university students [61]. Furthermore, although to a lesser extent, the type of institution (public or private) has been identified as an influential factor in satisfaction with the teaching–learning process, with university students from public institutions showing greater satisfaction [87].

In relation to satisfaction with the educational infrastructure, there are multiple studies that have empirically verified that facilities are indicated as a determining element for satisfaction by students from different areas of knowledge and in different countries, taking into account various aspects: library, communal areas, computers, classroom spaces, number of students per class, accommodation services, restaurant and sports, among others [78,79,88]. Regarding satisfaction with the educational infrastructure, according to gender, some studies do not find statistically significant differences [79,89], while others report that women are more satisfied than men [73]. Regarding the type of institution, the majority of research shows greater satisfaction with the infrastructure in private institutions [75,76,90]. However, in other investigations, it should be noted that it was students from public institutions who reported greater satisfaction with aspects related to infrastructure, such as library facilities or technological resources [87]. Likewise, some studies highlight an inverse relationship between the academic year and satisfaction with the educational infrastructure, with students in more advanced academic years showing the least satisfaction with educational infrastructure [65,73].

Taking into account the aforementioned, together with the fact that in Brazil there is little scientific production on university learning, and the satisfaction of students has still not been explored enough [91], interest in investigating these aspects is corroborated. Therefore, the first objective of this research is to evaluate the learning of Brazilian university students considering study techniques (study place and conditions, study organization and learning strategies), satisfaction with the teaching–learning process and with the educational infrastructure based on three variables: gender, institution of higher education and, the academic year of the Degree of Pedagogy students in the city of São Luís-Maranhão (Brazil). The second objective is to analyze the influence of age, gender, academic year, and institution on the general satisfaction of said students with respect to the educational infrastructure, the teaching–learning process and the study techniques used.

## 2. Materials and Methods

### 2.1. Participants

The sample consisted of 560 Brazilian students of the Pedagogy Degree (90.36% women and 9.64% men), with a minimum age of 16 and a maximum of 56 years old (M = 26.26; SD = 8.19). As can be seen, the Pedagogy Degree is studied mostly by women. The minimum age for starting university studies in Brazil is 16 years old. However, it is common for the age to be very diverse since students can start their studies later, because they did not start at a younger age, or because it is taken as a second degree.

These students were in each of the four academic years that make up the degree, that is, 1st year (*n* = 150; 26.8%), 2nd year (*n* = 140; 25.0%), 3rd year (*n* = 127; 22.7%) and 4th year (*n* = 143; 25.5%). They also attended classes in person at three higher education institutions in São Luís-Maranhão (Brazil): Universidade Estadual do Maranhão (UEMA) (*n* = 153; 27.3%), Universidade Federal do Maranhão (UFMA) (*n* = 235; 42%) and Faculdade Pythagoras (FP) (*n* = 172; 30.7%). The first two institutions are public and the last is private. Table 1 presents the description of the participants according to these variables.

Regarding the way students access university, 55.66% opted for the National High School Exam while the rest (44.4%) accessed after passing a specific exam at the university to which they aspired.

Ethnic–racial diversity in the sample is very wide, as occurs in the Brazilian population. In this regard, although the majority are mulattoes (57.53%), there were also whites (20.15%), blacks (19.24%), Asians (2.54%) and indigenous people (0.54%). Likewise, 50.2% did not work, while 49.8% did. In the latter, 30.1% worked in the education sector and 19.7% in other sectors.

### 2.2. Instruments

First of all, a section on sociodemographic data was included, such as age, gender, place of birth, institution of higher education, academic year and semester, shift, access route to university, etc.

Secondly, two questionnaires were used, one aimed at evaluating student satisfaction with respect to the educational infrastructure and the teaching–learning process and the other at evaluating the study techniques used by students.

#### 2.2.1. Satisfaction Questionnaire with the Educational Infrastructure and the Teaching–Learning Process

A questionnaire was designed based on different studies that focus on the relevance of educational facilities in higher education [70,78,79,81,92] as well as the teaching–learning process [6,8,93,94,95].

This questionnaire, made up of 63 items, was submitted to content validation, using the expert judgment technique. Ten specialists from different areas of higher education participated (Education, Psychology and Educational Research Methods) who were asked to respond on a Likert-type scale from 1 to 4, on the clarity of the writing and the relevance of the content of the items. The criteria established by Barbero [96] for inter-judge agreement were followed, that is:

- That the mean value of each item was equal to or greater than 2.5.
- Pay attention to the median value, as the value of the item.
- The 50th percentile (p. 50) should obtain values equal to or greater than 2.5.
- An ambiguity coefficient was established, which was intended to measure dispersion in the judges’ agreement, using the interquartile range as a criterion. Thus, if the difference between the 75th percentile (p.75) and the 25th percentile (p.25) was equal to 0 or 1, the item was accepted and/or slightly modified; If the difference was between 1 and 2, the item was revised and reformulated; whereas, if it was higher than 2, it was understood that the dispersion was high among the judges’ criterion, so the item was rejected.

After this process, four items were reviewed and their wording was modified, depending on the coefficient of ambiguity obtained (between 1.25 and 1.50) and the comments provided by the judges.

Subsequently, a pilot test was carried out with 15 students of the Pedagogy Degree from a higher education institution with the same characteristics necessary for the sample. This test also permitted observation of the time taken to answer the questions, as well as perceiving comprehension difficulties. Observations of the students regarding any terms not understood or the wording of some items were collected, proceeding to slight change in the wording of three items and inclusion of the response options regarding satisfaction with the educational infrastructure “Does not exist” and “I do not know”.

The final questionnaire was subsequently made up of 63 items structured in six categories: support infrastructure—external services, for example, banks (4 items); support infrastructure—internal services, such as conference or auditorium spaces (17 items); library resources, for instance, hard copies physically available in the library, that is, books, journals, dictionaries, encyclopedias, etc. (10 items); classroom facilities, for example, the size of the classroom (8 items); technological resources, such as computer rooms (7 items); teaching–learning process, for instance, teaching methodologies (17 items).

It had to be answered following a four-point Likert-type scale, where 1 = Totally dissatisfied and 4 = Totally satisfied. Likewise, regarding satisfaction with the educational infrastructure, the options “NE = Does not exist” and “NK = I do not know” were included.

The reliability of the questionnaire, evaluated using the Cronbach’s alpha internal consistency index, was 0.926.

#### 2.2.2. Study Techniques Questionnaire

A linguistic and cultural adaptation to the Brazilian context of the Study Techniques Questionnaire by Herrera and Gallardo [97] was used. In order to carry this out, indications outlined by the International Test Commission [98] were followed. These criteria have been widely used in the adaptation of tests [99]. In this regard, there was the participation of six Brazilian experts (three psychologists and three pedagogues), fluent in Spanish. They made the linguistic and cultural adaptation of the questionnaire, using a direct and inverse translation of the original instrument. Likewise, they attended to its content validity through the same procedure indicated in the previous questionnaire.

On the other hand, the test was also passed in a pilot study to 15 Pedagogy students and the wording of three of the items was slightly modified for a better understanding of them.

The final questionnaire was made up of 65 items, which referred to three categories: study place and conditions, for example, “Study in my bedroom” (17 items); organization of the study, for instance, “I usually plan the time that I am going to dedicate to the study” (15 items) and learning strategies, for example, “When I study, I relate the contents of the subject with those of other subjects” (33 items).

The students were asked to respond to a four-point Likert-type scale, where 1 = Never and 4 = Always.

The original version, in Spanish, of the Study Techniques Questionnaire has a reliability of 0.720 [100]. In this study, an internal consistency index of 0.828 was found.

### 2.3. Procedure

#### 2.3.1. Data Collection

Firstly, the approval of this study was obtained from the Brazilian Ethics Committee, following the ethical standards for the development of research with human beings, which was approved with reference code No. 3042080. Secondly, meetings were held with the heads of the six IHEs that offer the Pedagogy Course in the city of São Luís, presenting the research project, the data collection instruments and clarifying any possible doubts that might arise. Of the six institutions, three gave a positive response. Therefore, thirdly, a legal representative of each participating institution signed a formal authorization of the same, in which it was indicated, among other aspects, that the institutions could request more information regarding the study, authorizing the disclosure of data for scientific/academic purposes, respecting institutional secrecy and, in addition, that they would provide the requested results.

Once the different institutional authorizations had been obtained, the students were informed of the main objectives of the research and the instruments were designed. Those students who signed the Informed Consent were part of the sample. Likewise, the Informed Consent established that it was an anonymous, voluntary participation, without financial compensation and that they could stop their participation at any time. In addition, it was specified that the results of the study could be published in scientific/academic media.

The information collection instruments were applied in the second semester of the academic year at the beginning of the class schedule of a subject, in order to avoid major interruptions in the routine. Previously, a brief explanation of the instruments was provided, and they were read to resolve any questions. The mean time required to answer both instruments was 20–30 min.

#### 2.3.2. Analysis of Data

For data analysis, the statistical program IBM SPSS Statistics version 25 was used. Before starting with the different statistical analyses based on the research objectives, we proceeded to determine if the data fulfilled the normality criterion (Gaussian distribution), as well as the homogeneity of variances, in order to implement parametric or nonparametric statistical analysis tests.

To determine the normal distribution of the data, the Kolmogorov–Smirnov test was used, obtaining significant values for all items of the questionnaire, *p* < 0.001. Therefore, the Gaussian distribution was not satisfied. On the other hand, the homoscedasticity or homogeneity of variances was calculated. In the case of the independent variables gender (male vs. female) and type of IHE (public vs. private), the Levene statistic was used with the *t*-test for independent samples, not reaching the level of significance, *p* > 0.05, while for the variable grade (1st to 4th) the analysis of variance was used. No statistically significant results were obtained. Consequently, the homogeneity or equality of variances between the comparison groups was fulfilled.

In light of these results, some authors indicate that when normality is not fulfilled, nonparametric tests must be used [101]. However, given the size of the sample and that the requirement of homogeneity of variances was met, parametric statistical analysis tests were used, in the direction pointed out by different authors [102,103].

The mean score for each category of the two questionnaires was calculated. Likewise, the analysis of variance was used for the analysis based on the variables gender, type of IHE and academic year. The effect size was also analyzed, through the partial eta^2^ test, considering the following indices: *η^2^_p_* = 0.01 (small effect); *η^2^_p_* = 0.06 (medium effect); *η^2^_p_* = 0.14 (large effect) [104]. For the analysis of the effect size in the variables with two levels, Cohen’s d was used. In the case of the academic year variable, with four levels, the selected statistic for post-hoc comparisons was the Bonferroni test.

To respond to the second research objective, the mean score for satisfaction with the educational infrastructure, satisfaction with the teaching–learning process and study techniques was calculated. A linear regression analysis was developed in which, as a dependent variable, each of these variables were entered; and age, gender, grade, and type of IHE were entered as independent variables.

## 3. Results

### 3.1. Total Results

Table 2 shows the descriptive statistics obtained in each of the categories that make up the two evaluation instruments used.

In general, the score achieved in satisfaction with the different elements raised was average. As can be seen, the highest scores were those related to satisfaction with classroom facilities (*M* = 2.77, *SD* = 0.63) and with the teaching–learning process (*M* = 2.70, *SD* = 0.52), while the lowest ones refer to the place and study conditions (*M* = 2.12, *SD* = 0.31) as well as the technological resources (*M* = 2.20, *SD* = 0.61).

### 3.2. Analysis Based on the Variables Gender, Type of IHE and Academic Year

Table 3 presents the results of the three analysis of variance implemented based on the comparison variables.

No statistically significant differences were found based on gender, although there were on the type of IHE and the academic year. The results in each category of the questionnaires are shown in Table 4.

Satisfaction in public IHEs was lower than in private ones with respect to the support infrastructure—external services (*M_public_* = 2.33, *SD* = 0.43; *M_private_* = 2.46, *SD* = 0.48; d = −0.28), support infrastructure—internal services (*M_public_* = 2.22, *SD* = 0.45; *M_private_* = 2.42, *SD* = 0.49; d = −0.42), library resources (*M_public_* = 2.36, *SD* = 0.62; *M_private_* = 2.77, *SD* = 0.66; d = −0.64), the technological resources (*M_public_* = 2.14, *SD* = 0.55; *M_private_* = 2.37, *SD* = 0.71; d = −0.36) and the teaching–learning process (*M_public_* = 2.68, *SD* = 0.47; *M_private_* = 2.86, *SD* = 0.62; d = −0.33). In the same direction, differences were found in study techniques in relation to the organization of the study (*M_public_* = 2.32, *SD* = 0.36; *M_private_* = 2.44, *SD* = 0.46; d = −0.29) and learning strategies (*M_public_* = 2.58, *SD* = 0.33; *M_private_* = 2.67, *SD* = 0.39; d = −0.25).

By academic year, post-hoc comparisons using the Bonferroni test showed that satisfaction with the support infrastructure—external services was higher in the first year (*M* = 2.48, *SD* = 0.44) compared to the third (*M* = 2.32, *SD* = 0.42; *p* = 0.014) and fourth (*M* = 2.30, *SD* = 0.47; *p* = 0.003). Satisfaction with the support infrastructure—internal services was higher in first (*M* = 2.46, *SD* = 0.48) compared to second (*M* = 2.22, *SD* = 0.45; *p* = 0.000), third (*M* = 2.21, *SD* = 0.40; *p* = 0.000) and fourth (*M* = 2.20, *SD* = 0.48; *p* = 0.000). Also satisfaction with the library resources in the first year (*M* = 2.75, *SD* = 0.64) compared to second (*M* = 2.40, *SD* = 0.66; *p* = 0.000), third (*M* = 2.38, *SD* = 0.54; *p* = 0.000) and fourth (*M* = 2.38, *SD* = 0.68; *p* = 0.000). Satisfaction with the classroom facilities, again, was higher in first (*M* = 2.99, *SD* = 0.59) compared to second (*M* = 2.73, *SD* = 0.62; *p* = 0.002), third (*M* = 2.65, *SD* = 0.60; *p* = 0.000) and fourth (*M* = 2.69, *SD* = 0.65; *p* = 0.000). Satisfaction with technological resources, likewise, was higher in the first (*M* = 2.40, *SD* = 0.58) than in the second (*M* = 2.16, *SD* = 0.62; *p* = 0.005), third (*M* = 2.06, *SD* = 0.52; *p* = 0.000) and fourth (*M* = 2.19, *SD* = 0.67; *p* = 0.019). For its part, the learning strategies used by the students were significantly higher in the first year (*M* = 2.64, *SD* = 0.34) compared to the third (*M* = 2.53, *SD* = 0.32; *p* = 0.019).

### 3.3. Predictive Analysis

Firstly, a linear regression analysis was carried out in which satisfaction with the infrastructure was introduced as the dependent variable and gender, type of IHE, grade and age as predictive variables (see Table 5). The model was significant, *F* = 14.921, *p* = 0.000. Durbin–Watson’s *d* test showed that there was no autocorrelation in the data (*d* = 1505). Values of the Durbin–Watson test between 1.5 and 2.5 indicate that the data are not correlated [105]. In addition, the variance inflation factor (VIF) obtained values lower than 5, so multicollinearity was not present [106,107].

The type of IHE predicted satisfaction with the infrastructure and the academic year, in the latter case, the prediction was negative.

Secondly, a linear regression analysis was carried out in which satisfaction with the teaching–learning process was introduced as a dependent variable (see Table 6). The model was significant, *F* = 6.449, *p* = 0.000. The data were not correlated (*d* = 1658) and no multicollinearity was found.

The type of IHE predicted satisfaction with the teaching–learning process and, to a lesser extent and negatively, age.

The results of the linear regression analysis in which the dependent variable introduced was the one related to the study techniques is presented in Table 7.

The model was significant (*F* = 3260, *p* = 0.012), and the data were not correlated (*d* = 1959), in addition to not finding multicollinearity.

The type of IHE was the variable that best predicted the study techniques used. Age was also a negative predictor.

## 4. Discussion

The first objective of this research was to evaluate the learning of Brazilian university students considering study techniques, satisfaction with the teaching–learning process and with the educational infrastructure, based on three variables: gender, institution of higher education and the academic year. As can be seen in the results, no statistically significant differences were obtained regarding gender. Although the Pedagogy Degree is studied mostly by women, this is an important result since women in Latin America and the Caribbean have historically faced a situation of inequality with respect to access to education and since the positive relation between the level of education and salary has been highlighted [108]. This result agrees with those of previous authors, who found no differences in learning self-regulation strategies [56,57,58,59], in satisfaction with the teaching–learning process [86] or even in happiness [109] depending on gender. These results differ, however, from other studies that showed how women used more self-regulation strategies than men [50,53,55], by using more metacognitive testing, elaboration, organization and processing tools [52]; as well as that women are those who show a lower level of satisfaction with the teaching–learning process [74]. The fact that no gender differences were found in the present study may be due to the university education becoming increasingly egalitarian, a transformation that has taken place in parallel with the historical–social changes in terms of the perception of their rights and their autonomy in the various areas of their lives [110]. In fact, in 2015, for the first time in a country like Spain, the percentage of female graduates was 56%, very close to the OECD average (57%) and the EU22 (59%) [111].

However, other authors point out that the influence of this variable is mediated by other factors such as age [61], which will be discussed later, or the type of degree they are studying [60]. It should be noted that women continue to be underrepresented in science, technology, engineering and mathematics (STEM) careers, especially in GEMP careers (geosciences, engineering, economics, math/computer science, and physical science), which are the careers that continue to generate the highest income [112,113].

Regarding the institution, statistically significant differences were found in the type of IHE, both in study techniques, and in satisfaction with the infrastructure and teaching–learning processes. In all cases, it was private institutions, which scored the highest. With respect to satisfaction with the infrastructure, there were statistically significant differences in the type of IHE in all the variables (support infrastructure—external services, support infrastructure—internal services, library resources, technological resources), except in classroom facilities. This last result is very relevant since some authors have highlighted the importance of classroom facilities—the most basic facilities—in the general satisfaction with the infrastructure of the students [10]. However, in other resources, for example, the library, which is more dependent on financial resources, there were statistically significant differences depending on the type of IHE and this is also a very essential variable for student satisfaction [78]. This situation has already been highlighted in other studies, where private education students report receiving more guidance and better treatment from their teachers [114]. It should be noted that in Brazil, there is still a marked difference between private and public universities. As some authors state [115], legislators have yet to explore and implement more tools to monitor and improve the quality of the Brazilian public education system, bearing in mind the data regarding student satisfaction or their employability. This would improve, in turn, the general image of these institutions [91]. The system of HTVE (Higher Technical and Vocational Education) colleges, which are mainly public, still faces important challenges in Brazil, such as the standardization of studies, poor coordination with the labour market or the high dropout rate of students [115]. The fact that the studies are not yet sufficiently standardized may be one of the variables that have been able to influence the lower satisfaction of students from public institutions with the teaching–learning process and that has also been able to influence satisfaction with their own study techniques. However, this situation and the results obtained in the present study could be reversed in the future, since in a few years, the number of HTVE colleges is growing exponentially and they have been installed in more than 10% of Brazilian cities [116]. This expansion implies public policies that promote economic and social development and offer greater opportunities, especially for the most vulnerable, to study, for employability and to increase satisfaction with these institutions [116,117,118].

Another of the most influential variables in study techniques and student satisfaction with the infrastructure is the academic year. Specifically, it is first-year students who most positively value the aspects related to the infrastructure of their university (in all subscales: external services, internal services, library resources, classroom facilities and technological resources) and their study techniques (especially their learning strategies). Regarding infrastructure, this result contrasts with other studies where it is graduates, who have completed the entire degree, who best value infrastructure compared to students who continue studying at university [119]. On the other hand, some previous research indicates that it is the students of higher levels who develop more and more effective learning strategies than those used by students of lower grades [51,54]. Other research, however, supports a detriment of effort in higher levels compared to lower ones [52], which agrees with the results of the present study, or even establishes that there is no clear evidence that students at higher levels develop strategies for deeper or more elaborate learning [62].

The second objective of this study was to analyze the influence of age, gender, academic year, and institution on the general satisfaction of students with respect to the educational infrastructure, the teaching–learning process and the study techniques used. Again gender did not present statistically significant values; while the type of IHE and the academic year showed predictive values in satisfaction with the infrastructure; the type of IHE and the age they did with respect to the teaching–learning process; and the type of IHE and the age in terms of study techniques. As previously discussed, an inverse relationship was observed in the academic year, that is, the higher the grade, the less satisfaction with the infrastructure. However, the academic year was not found to have a predictive value in Study Techniques. This same inverse relationship occurred in the age variable (the older the lower score) in satisfaction with the teaching–learning processes and study techniques, so it may be deduced that, although the academic year influences study techniques, this influence may be mediated by the age [61] or the maturity of the student when using them.

This research provides valuable information on the processes of self-regulation and external regulation of learning and satisfaction with these aspects in Brazilian students, nevertheless, not without limitations and possible improvements. Future research should clarify some of the issues raised here. Some of these are the reasons why students in higher academic years and ages use fewer study techniques or are more dissatisfied with the infrastructure or also with the teaching–learning processes. The fact that at higher levels, less study techniques are used instead of more, can be seen as a contradiction, so it would be necessary to include other variables, possibly mediators, that abound in this direction. One might wonder if students abandon some of these techniques throughout their careers because they are not entirely useful or do not have a direct impact on their performance. Following this approach, it could be investigated, for example, whether or not there is direct teaching of these techniques by teachers or if other traditional study techniques are added that might improve student’s concentration, performance and help to cope with academic life, such as the practice of mindfulness [120]. Another aspect to explore might be the influence of the COVID-19 pandemic and the new university setting, with a greater presence of virtual formats, on student satisfaction, especially with teaching–learning processes.

## 5. Conclusions

The most relevant corollaries of the present study refer, firstly, to the importance of evaluating the factors that influence self-regulation and external regulation of learning and the satisfaction of university students with these aspects.

Firstly, it has been shown that the type of IHE affects both satisfaction with the infrastructure and with the teaching–learning processes, as well as study techniques; and it is one of the greatest predictors of student satisfaction with these variables.

Secondly, the academic year is another of the predictors of satisfaction with the infrastructures, with those in the first year being the most satisfied with respect to those in higher academic years. This same first-year student is the one that uses the most study techniques, specifically, learning strategies. However, this last aspect may be mediated by age, as this is one of the variables with the highest predictive value in study techniques, as well as in Satisfaction with the teaching–learning processes. The influence of age was also inverse, that is, the older the age, the lower the use of study techniques and the lower satisfaction with the teaching–learning processes.

Finally, no statistically significant differences were found in the scores of the scales based on gender, nor was it a variable with predictive value in any of them. This result shows that, in recent years, “The University has therefore managed to become an agent of transformation that promotes social development from the perspective of justice, solidarity and equal opportunities” (p. 11, [121]). Although these advances do not imply that there is still no path to traverse in pursuit of an education increasingly free of gender stereotypes.

Despite the importance of the results obtained in this study, especially in the comparison of public and private universities in Brazil, being a sample limited to a specific career, we cannot generalize for the general context of Brazil.

## Figures and Tables

**Table 1:** Characteristics of the participants of this study.

IHE	Academic Year	Gender			Edad			
		Male	Female	Total	Minimum	Maximum	*M*	*SD*
UEMA	1.º	2	43	45	16	35	21.73	4.42
	2.º	2	31	33	18	38	22.78	5.12
	3.º	3	30	33	19	56	26.74	10.24
	4.º	3	39	42	19	37	23.40	3.44
UFMA	1.º	6	53	59	17	40	22.75	4.95
	2.º	6	71	77	18	56	26.10	8.71
	3.º	11	42	53	19	49	28.52	9.55
	4.º	9	37	46	20	53	28.15	8.96
FP	1.º	3	43	46	18	48	27.37	8.73
	2.º	3	27	30	19	49	27.35	7.42
	3.º	4	37	41	20	46	30.15	7.48
	4.º	2	53	55	20	50	29.81	7.89
Total		54	506	560	16	56	26.26	8.19

Note. IHE = Institution of Higher Education; UEMA = State University of Maranhão; UFMA = Federal University of Maranhão; FP = Faculdade Pythagoras; M = mean; SD = standard deviation.

**Table 2:** Descriptive statistics in each category of the questionnaires.

	Categories	*N*	Minimum	Maximum	*M*	*SD*
Satisfaction with the educative infrastructure	Support infrastructure—external services	560	1.00	4.00	2.37	0.44
	Support infrastructure—internal services	560	1.00	4.00	2.28	0.47
	Library resources	560	1.00	4.00	2.48	0.65
	Classroom facilities	560	1.00	4.00	2.77	0.63
	Technological resources	560	1.00	4.00	2.20	0.61
Satisfaction with the teaching–learning process	Teaching–learning process	560	1.00	4.00	2.70	0.52
Study techniques	Study place and conditions	560	1.00	4.00	2.12	0.31
	Study organization	560	1.00	4.00	2.35	0.39
	Learning strategies	560	1.00	4.00	2.60	0.35

Note. *M* = mean; *SD* = standard deviation.

**Table 3:** Main effects of the comparison variables.

Variables	F	*p*	Eta^2^ p
Gender	1.470	0.156	0.024
Type of IHE	8.272 ***	0.000	0.119
Academic year	27.000 ***	0.000	0.041

*** *p* < 0.001.

**Table 4:** Analysis of variance in each category according to gender, type of IHE and academic year.

		Gender			Type of IHE			Academic Year		
	Categories	F	*p*	Eta^2^ p	F	*p*	Eta^2^ p	F	*p*	Eta^2^ p
Satisfaction with the educative infrastructure	Support infrastructure-External services	3.029	0.082	0.005	8.845 **	0.003	0.016	4.959 **	0.002	0.026
	Support infrastructure-Internal services	0.002	0.966	0.000	22.454 ***	0.000	0.039	10.807 ***	0.000	0.055
	Library resources	1.914	0.167	0.003	48.385 ***	0.000	0.080	11.645 ***	0.000	0.059
	Classroom facilities	0.101	0.750	0.000	2.049	0.153	0.004	8.906 ***	0.000	0.046
	Technological resources	1.277	0.259	0.002	17.079 ***	0.000	0.030	7.677 ***	0.000	0.040
Satisfaction with the teaching–learning process	Teaching–learning process	1.108	0.293	0.002	19.601 ***	0.000	0.034	2.134	0.095	0.011
Study techniques	Study place and conditions	0.729	0.394	0.001	0.061	0.805	0.000	1.337	0.261	0.007
	Study organization	0.064	0.800	0.000	10.881 **	0.001	0.019	1.925	0.124	0.010
	Learning strategies	2.309	0.129	0.004	6.755 *	0.010	0.012	3.522 *	0.015	0.019

*** *p* < 0.001, ** *p* < 0.001, * *p* < 0.05.

**Table 5:** Regression analysis regarding satisfaction with the infrastructure.

Variables	Non-Standardized Coefficients		Standardized Coefficients	*t*	*p*	Colinearity Tests	
	B	SE	Beta			TI	VIF
Gender	−0.048	0.067	−0.029	−0.711	0.477	0.983	1.018
Type of IHE	0.231	0.042	0.233	5.560 ***	0.000	0.948	1.055
Academic year	−0.092	0.017	−0.230	−5.527 ***	0.000	0.961	1.040
Age	−0.003	0.002	−0.050	−1.186	0.236	0.918	1.089

*** *p* < 0.001; *SE* = standard error; TI = tolerance index; VIF = variance inflation factor.

**Table 6:** Regression analysis regarding satisfaction with the teaching–learning process.

Variables	Non-Standardized Coefficients		Standardized Coefficients	*t*	*p*	Colinearity Tests	
	B	SE	Beta			TI	VIF
Gender	0.016	0.079	0.008	0.198	0.843	0.983	1.018
Type of IHE	0.222	0.049	0.197	4.564 ***	0.000	0.948	1.055
Academic year	−0.035	0.020	−0.076	−1.786	0.075	0.961	1.040
Age	−0.006	0.003	−0.086	−1.974 *	0.049	0.918	1.089

*** *p* < 0.001, * *p* < 0.05.

**Table 7:** Regression analysis regarding study techniques.

Variables	Non-Standardized Coefficients		Standardized Coefficients	*t*	*p*	Colinearity Tests	
	B	SE	Beta			TI	VIF
Gender	0.016	0.041	0.017	0.396	0.692	0.983	1.018
Type de IHE	0.076	0.025	0.131	3.013 **	0.003	0.948	1.055
Academic year	−0.007	0.010	−0.032	−0.735	0.463	0.961	1.040
Age	−0.003	0.001	−0.095	−2.147 *	0.032	0.918	1.089

** *p* < 0.01, * *p* < 0.05.

## Data Availability

The data presented in this study are available on request from the corresponding author. The data are not publicly available due to privacy and ethical reasons.
